# Association between Albumin-Corrected Anion Gap and Mortality in Patients with Cardiogenic Shock

**DOI:** 10.31083/j.rcm2506226

**Published:** 2024-06-21

**Authors:** Meng Yuan, Lei Zhong, Jie Min, Jianhong Lu, Lili Ye, Qikai Shen, Beiping Hu, Haiying Sheng

**Affiliations:** ^1^Department of Intensive Care Unit, Huzhou Central Hospital (The Fifth School of Clinical Medicine of Zhejiang Chinese Medical University), Affiliated Central Hospital Huzhou University, 313000 Huzhou, Zhejiang, China; ^2^Department of Cardiology, Huzhou Central Hospital (The Fifth School of Clinical Medicine of Zhejiang Chinese Medical University), Affiliated Central Hospital Huzhou University, 313000 Huzhou, Zhejiang, China; ^3^Department of Catheterization-Room, Huzhou Central Hospital (The Fifth School of Clinical Medicine of Zhejiang Chinese Medical University), Affiliated Central Hospital Huzhou University, 313000 Huzhou, Zhejiang, China

**Keywords:** cardiogenic shock, albumin-corrected anion gap, mortality, prognosis, MIMIC-IV

## Abstract

**Background:**

Cardiogenic shock (CS) is a critical illness with a high mortality rate in clinical practice. Although some biomarkers have been found to be associated with mortality in patients suffering from CS in previous studies. The albumin-corrected anion gap (ACAG) has not been studied in depth. Our study aimed to explore the relationship between ACAG and mortality in patients with CS.

**Methods:**

All baseline data was extracted from Medical Information Mart for Intensive Care-IV version: 2.0 (MIMIC-IV). According to the prognosis at 30 days of follow-up, they were divided into survivors and non-survivors groups. The survival curves between the two groups were drawn using the Kaplan-Meier method and the log-rank test. Valid factors were selected using the least absolute shrinkage and selection operator (LASSO) logistic analysis model. Analysis was performed to investigate the relationship between mortality and all enrolled patients using restricted cubic spline (RCS) and Cox proportional hazards models. Receiver operating characteristic (ROC) curves were used to assess the predictive ability of ACAG. Evaluation of final result stability using sensitivity analysis.

**Results:**

839 cases were selected to meet the inclusion criteria and categorized into survivors and non-survivors groups in the final analysis. The ACAG value measured for the first time at the time of admission was selected as the research object. Kaplan-Meier (K-M) survival curves showed that cumulative 30- and 90-day survival decreased progressively with elevated ACAG (*p* < 0.001), and multifactorial Cox regression analyses showed ACAG to be an independent risk factor for increased 30- and 90-day mortality in patients suffering from CS (*p* < 0.05). RCS curves revealed that all-cause mortality in this group of patients increased with increasing ACAG (χ^2^ = 5.830, *p* = 0.120). The ROC curve showed that the best cutoff value for ACAG for predicting 30-day mortality in patients with CS was 22.625, with a sensitivity of 44.0% and a specificity of 74.7%. The relationship between ACAG and CS short-term mortality remained stable in all sensitivity analyses (All *p* < 0.05).

**Conclusions:**

The ACAG is an independent risk factor for 30- and 90-day mortality in CS patients and predicts poor clinical outcomes in CS patients. According to our study, elevated ACAG at admission, especially when ACAG >20 mmol/L, was an independent predictor of all-cause mortality in CS.

## 1. Introduction

Cardiogenic shock (CS) is a condition in which a significant decrease in cardiac output is inadequate to perfuse the tissues and cardiac pump failure causes severe multiple organ dysfunction [1]. Despite modern cardiology’s rapid development, the cardiogenic shock field has progressed slowly in recent years, and its short-term mortality has not changed, still reaching 40–50% [2]. Most of the patients are seriously ill and need to be admitted to the intensive care unit (ICU) for treatment, the treatment cost is high, and the prognosis is poor [3]. Although many prognostic predictive biological indicators for CS have been identified [4], more research is needed to determine whether they are fully applicable. Predictors of the early prognosis of CS deserve further exploration and study.

Tissue hypoxia can cause metabolic disorders when CS occurs, and acidosis often predicts a worse prognosis in CS [5]. The anion gap (AG) is commonly used to judge acid-base balance in clinical practice. AG has also been used as a predictor of mortality in some diseases, and AG levels are associated with adverse prognoses, for instance acute kidney injury (AKI), acute ischemic stroke, and coronary artery disease [6, 7, 8]. Patients with heart failure, acute myocardial infarction (AMI), or cardiopulmonary arrest may develop CS [9]. Xu *et al*. [10] found that the increased 30 days, 180 days, and 1-year mortality in AMI patients were both affected by increased AG. At the same time, Tang *et al*. [11] also pointed out that the 90-day mortality in patients with congestive heart failure was related to AG. It is worth mentioning that, previous studies have shown that AG can be used as a predictor of all-cause mortality in CS [12]. However, AG is affected by many factors, especially the prevalence of hypoalbuminemia in critically ill patients, which can easily lead to AG errors. Therefore, the concept of albumin-corrected anion gap (ACAG) was introduced, and one study found that the predictive value of ACAG for mortality was higher than AG in severe sepsis patients [13]. Research has shown that both AG and ACAG are associated with recovery of spontaneous circulation, but ACAG is more effective than AG in predicting recovery of spontaneous circulation in patients with cardiorespiratory arrest [14]. However, there are still no reports on whether the predictive relationship between ACAG and CS is clear.

Hence, this study mainly explores whether the ACAG level at the time of admission to the ICU is a predictor of 30-day and 90-day all-cause mortality in CS patients, assists doctors in evaluating the condition in clinical practice, and provides a basis for early prognostic intervention.

## 2. Materials and Methods

### 2.1 Database

All data in this study come from the Medical Information Mart for Intensive Care-IV version: 2.0 (MIMIC-IV) database (2008 to 2019) [15], which has approval from through Massachusetts Institute of Technology and Institutional Review Board of Beth Israel Deaconess Medical Center (BIDMC) and is deidentified according to Health Insurance Portability and Accountability Act Safe Harbor provision. This information is freely available and authentic. Two authors, Meng Yuan, and Lei Zhong, have completed the Collaborative Institution Training Program exam (Certification No. MY: 51168595; LZ: 53446653) and have database access to extract data.

### 2.2 Study Population

**Fig. 1.**
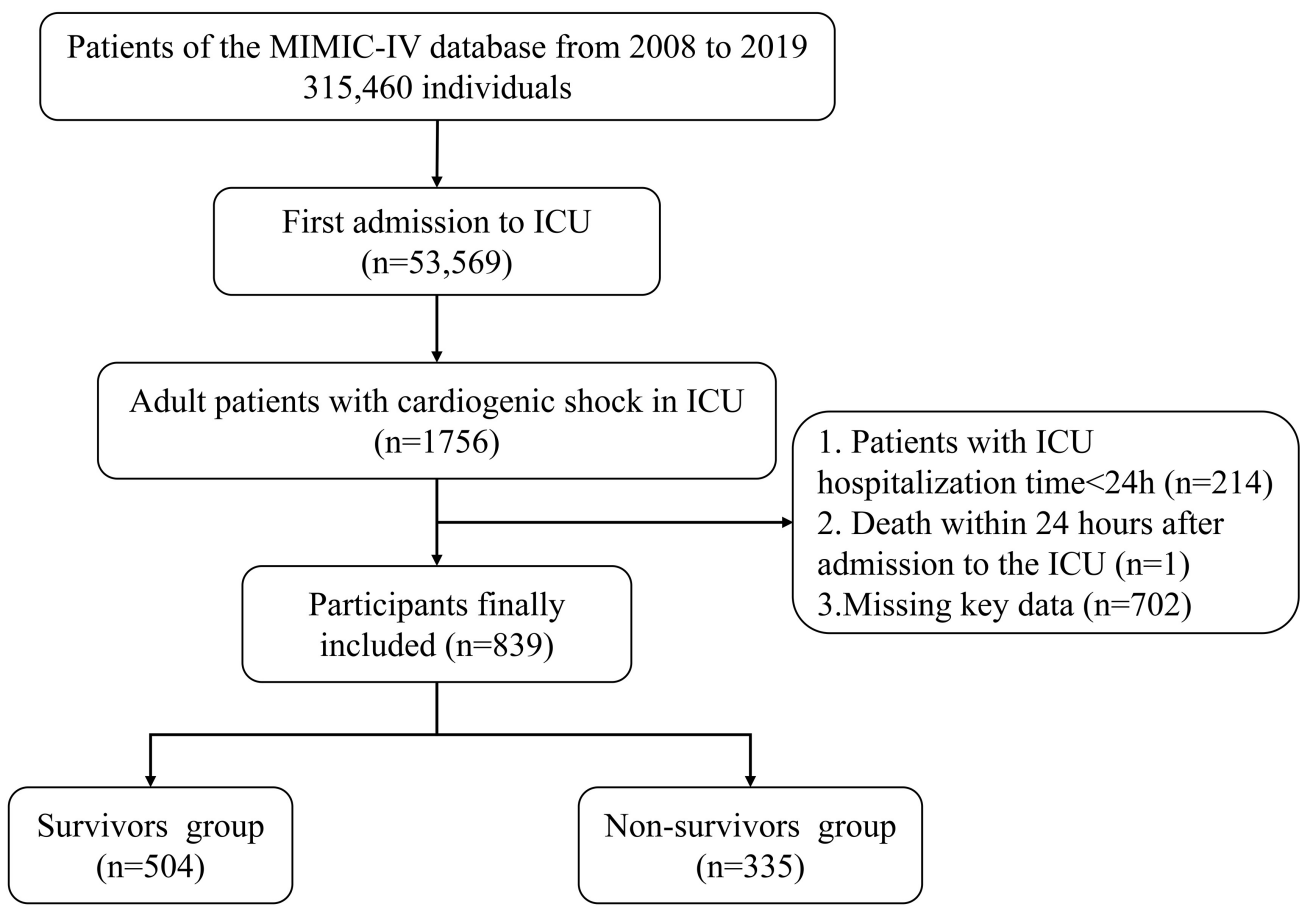
**The detailed process of data extraction. **ICU, intensive care unit; MIMIC-IV, Medical Information Mart for Intensive Care-IV version: 2.0.

The inclusion criteria were: (1) adult patients with cardiogenic shock in ICU (age ≥18 years); (2) albumin measured within 24 hours of ICU admission; (3) anion gap values available within 24 hours of ICU admission. This study only selected patients who met the above criteria and were admitted to the ICU for the first time, because the same patient may be repeatedly admitted to the ICU. In addition, patients with a hospital stay of less than 24 hours were excluded due to more missing key data (Fig. 1).

### 2.3 Data Extraction and Study Outcomes

Using structure query language (SQL) to enter the MIMIC-IV database, the patient information was identified and extracted according to the diagnostic code of CS in International Classification of Diseases (ICD)-9 and ICD-10 codes (78551, 99801, R570, T8111, T8111XA, T8111XD, T8111XS). Extracted variables included age, sex, laboratory parameters, comorbidities, severity scoring system, length of hospital stay, examination, treatments, and time to death. Laboratory parameters include hematocrit, albumin, hemoglobin, platelets, prothrombin time (PT), serum creatinine, white blood cell (WBC), blood glucose, blood urea nitrogen (BUN), potassium, alanine aminotransferase (ALT), etc. Severity scoring systems include sequential organ failure assessment (SOFA) score and Acute Physiology Score (APS) III. Examination and treatments that may affect the prognosis include the use of drugs such as norepinephrine, mechanical circulatory support such as intra-arterial balloon counterpulsation (IABP), tests such as coronary angiography, and more. All laboratory data must be measured within 24 hours of the patient’s admission to the ICU. The average value is taken if a certain item is tested multiple times within 24 hours after admission. The ACAG is calculated as: ACAG (mmol/L) = [4.4 – (albumin (g/dL))] × 2.5 + AG [16]. According to the ACAG level, it is divided into the normal ACAG group (12–20 mmol/L) and the high ACAG group (>20 mmol/L) [17]. The primary outcome of the study was 30-day all-cause mortality in CS patients, and the secondary outcome was 90-day all-cause mortality.

### 2.4 Statistical Analysis

The study used the Kolmogorov-Smirnov test to assess normal distribution of the continuous variable, and the variable was expressed in the form of mean ± standard deviation (SD), and the *t* test was used when the sample size is small and the overall standard deviation cannot be calculated. The medians of the interquartile range (IQR) were used when the continuous variable does not conform to the normal distribution, and variables were tested using the Wilcoxon rank-sum test. Categorical variables were expressed as numbers and percentages and they were tested using the Chi-square test. The patients were divided into two groups based on their ACAG levels: 12 ≤ ACAG ≤ 20 mmol/L and ACAG >20 mmol/L, which were calculated using the previous method [16]. The least absolute shrinkage and selection operator (LASSO) logistic analysis model approach was used to select the most useful predictive features from the primary data set. The optimal adjustment parameter (λ) for the final model was determined through 10-fold cross-validation. The optimal λ resulted in the selection of all non-zero parameters for further statistical analysis. The separated groups were compared using the Kaplan-Meier survival estimates were used to estimate the association between ACAG and risk of 30-day and 90-day mortality in intensive care patients with CS for each group. The Cox proportional hazards regression model was used to analyze the relationship between 30-day and 90-day mortality and ACAG of CS patients in the ICU, and to ensure the accuracy of the statistical results, the interference caused by other confounding factors was adjusted in the multiple regression analysis. The results were expressed as the hazard ratio (HR) with a 95% confidence interval (CI). Model 1 does not adjust any variable. In model 2, we adjusted for covariates including unequal baseline characteristics and clinically relevant factors (effective predictive characteristics obtained in the LASSO logistic analysis model). And we drew a curve between ACAG and 30-day and 90-day mortality of CS patients in the ICU using the restricted cubic spline (RCS) model to assess whether there was a linear relationship. To assess the predictive capability of ACAG, we utilized Receiver Operating Characteristics (ROC) curves. Sensitivity analysis was performed by discharging the relevant elements that might affect the outcome of the statistical analysis. To verify the stability of the results, we performed the multifactorial analysis again after excluding patients who were infused with albumin in the first 48 hours of admission to the intensive care unit (n = 832). In addition, the analysis was performed again after excluding patients with cirrhosis (n = 791), malignant tumours (MT) patients (n = 757), and patients with other severe liver diseases (n = 806). All data were analyzed using Stata 14.0 software (Stata Corp, College Station, TX, USA) and R software (version 4.2.3, R Foundation for Statistical Computing, Vienna, Austria). If the *p*-value < 0.05, the results are considered statistically significant.

## 3. Results

### 3.1 Baseline Characteristics

A total of 315,460 medical cases were reviewed, including 2547 adult patients with cardiogenic shock, of which 791 patients who were repeatedly admitted to the ICU were excluded, 214 patients with hospitalization time less than 24 h, 1 patient death within 24 h after admission to the ICU, and 702 patients cannot calculate ACAG due to missing key data such as albumin. A total of 839 patients were included, and they were divided into the survivors group (n = 504) and the non-survivors group (n = 335) according to their prognosis at 30 days of follow-up (Fig. 1). The age, AG, ACAG, WBC, PT, BUN, serum creatinine, SOFA, and APS III score of the non-survivors group were significantly higher than those of the survival group (*p* < 0.05). The albumin, hematocrit, and total hospitalization time of the survivors group were higher than those of the non-survivors group. The number of people with comorbidities including diabetes, AKI, chronic kidney disease (CKD), MT; congestive heart failure; ventricular fibrillation, and acute respiratory failure was higher in the non-survivors group than in the survivors group (*p* < 0.05). A higher number of individuals in the survivors group went through coronary arteriography (*p* = 0.001), while a greater number of individuals in the non-survival group utilized continuous renal replacement therapy and mechanical ventilation, along with norepinephrine, dopamine, and vasopressin (*p* < 0.05; Table 1).

**Table 1.** **Baseline characteristics of all study populations**.

Variables		Total	Survivors	Non-survivors	t/Z/χ^2^	*p*
		(n = 839)	(n = 504)	(n = 335)		
Baseline variables						
	Age (years)	69.37 ± 15.15	67.67 ± 15.23	71.93 ± 14.67	–4.024	<0.001
	Male, n (%)	483 (57.57)	300 (59.52)	183 (54.63)	1.976	0.160
Laboratory parameters						
	AG (mmol/L)	18.55 ± 5.35	17.74 ± 4.92	19.79 ± 5.74	–5.522	<0.001
	Albumin (g/L)	32.43 ± 6.11	33.27 ± 5.88	31.17 ± 6.23	4.934	<0.001
	ACAG (mmol/L)	21.45 ± 5.37	20.42 ± 4.88	22.99 ± 5.70	–6.989	<0.001
	WBC (×${\mathrm{10}}^{9}$/L)	12.8 (8.9, 17.3)	12.6 (8.7, 16.75)	13.1 (9.1, 18.2)	–1.720	0.085
	Hematocrit (%)	35.83 ± 7.58	36.50 ± 7.47	34.82 ± 7.58	3.148	0.002
	PLT (×${\mathrm{10}}^{9}$/L)	211 (152, 277)	215 (152.5, 279.5)	201 (150, 276)	1.173	0.241
	PT (s)	15.0 (12.8, 20.0)	14.35 (12.6, 19.15)	15.9 (13.6, 21.7)	–4.483	<0.001
	ALT (U/L)	52 (24, 141)	52 (23, 146.5)	52 (25, 138)	0.492	0.623
	AST (U/L)	93.0 (39.0, 270.0)	91.0 (37.0, 275.5)	94.0 (42.0, 263.0)	–0.008	0.994
	BUN (mmol/L)	10.324 (6.764, 16.732)	9.612 (6.052, 14.952)	12.46 (7.832, 19.224)	–4.659	<0.001
	Scr (µmol/L)	123.76 (88.40, 203.32)	123.76 (88.4, 176.8)	141.44 (106.08, 238.68)	–4.432	<0.001
	Glucose (mmol/L)	8.67 (6.56, 12.55)	8.44 (6.58, 11.69)	9.00 (6.50, 13.94)	–1.875	0.061
	Potassium (mmol/L)	4.64 ± 1.07	4.60 ± 1.08	4.70 ± 1.04	–1.258	0.209
	Total calcium (mmol/L)	2.09 ± 0.25	2.09 ± 0.26	2.09 ± 0.24	0.081	0.935
Examination and treatment [n (%)]						
	Coronary Arteriography	154 (18.36)	110 (21.83)	44 (13.13)	10.143	0.001
	Transthoracic echocardiography	375 (44.70)	221 (43.85)	154 (45.97)	0.366	0.545
	MV	635 (75.69)	354 (70.24)	281 (83.88)	20.353	<0.001
	IABP	179 (21.33)	117 (23.21)	62 (18.81)	2.656	0.103
	CRRT	130 (15.49)	52 (10.32)	78 (23.28)	25.839	<0.001
	Defibrillation	95 (11.32)	57 (11.31)	38 (11.34)	0.000	0.988
	Norepinephrine use	564 (67.22)	285 (56.55)	279 (83.28)	65.286	<0.001
	Dopamine use	196 (23.36)	104 (20.63)	92 (27.46)	5.240	0.022
	Vasopressin use	270 (32.18)	98 (19.44)	172 (51.34)	93.823	<0.001
Comorbidities [n (%)]						
	Hypertension	244 (29.08)	158 (31.35)	86 (25.67)	3.145	0.076
	Diabetes	296 (35.28)	162 (32.14)	134 (40.00)	5.441	0.020
	COPD	250 (29.80)	149 (29.56)	101 (30.15)	0.033	0.856
	CKD	280 (33.37)	143 (28.37)	137 (40.90)	14.192	<0.001
	Cardiac arrest	128 (15.26)	70 (13.89)	58 (17.31)	1.825	0.177
	AMI	372 (44.34)	229 (45.44)	143 (42.69)	0.617	0.432
	ARF	409 (48.75)	231 (45.83)	178 (53.13)	4.293	0.038
	AKI	690 (82.24)	390 (77.38)	300 (89.55)	20.411	<0.001
	VF	90 (10.73)	45 (8.93)	45 (13.43)	4.263	0.039
	MT	82 (9.77)	36 (7.14)	46 (13.73)	9.906	0.002
	CHF	629 (74.97)	396 (73.21)	233 (69.55)	8.723	0.003
Score system						
	APS III score	70.26 ± 29.39	60.79 ± 25.56	84.50 ± 29.07	–12.449	<0.001
	SOFA score	9.13 ± 4.30	8.09 ± 4.14	10.70 ± 4.06	–9.008	<0.001
TLOS (days)		9.92 (5.46, 17.21)	12.88 (7.79, 21.25)	6.38 (2.79, 11.92)	11.689	<0.001

AG, anion gap; PLT, platelets; PT, prothrombin time; ALT, alanine aminotransferase; AST, aspartate aminotransferase; APS, acute physiology score; ACAG, albumin corrected anion gap; BUN, blood urea nitrogen; WBC, white blood cell; Scr, serum creatinine; SOFA, sequential organ failure assessment; COPD, chronic obstructive pulmonary disease; CKD, chronic kidney disease; AKI, acute kidney injury; ARF, Acute respiratory failure; AMI, acute myocardial infarction; MT, malignant tumors; CHF, congestive heart failure; VF, ventricular fibrillation; MV, mechanical ventilation; CRRT, continuous renal replacement therapy; IABP, intra-arterial balloon counterpulsation; TLOS, total length of stay. *p* < 0.05 means a significant difference.

### 3.2 Between ACAG and 30-Day and 90-Day Mortality

**Fig. 2.**
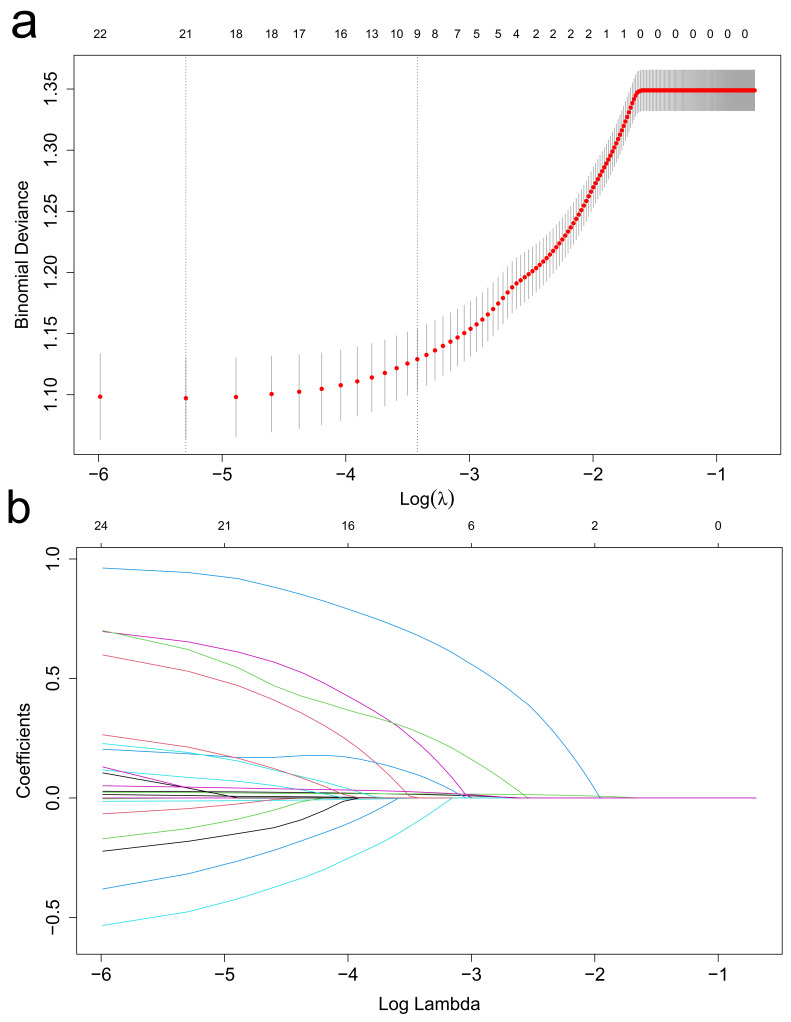
**The least absolute shrinkage and selection operator (LASSO) logistic regression model.** (a) To determine the minimum pass criterion for the final model, we used the adjustment parameter (λ) and plotted the area under the receiver operating characteristic (AUC) curve against log(λ) using a 10-fold cross-validation. Optimal values were identified by drawing dotted vertical lines at the minimum criteria and the 1 standard error of the minimum criteria (the 1-SE criteria). After the 10-fold cross-validation, the λ value of 0.0326 was calculated. (b) The vertical lines were drawn at the optimal values by using the 10-fold cross-validation, and the optimal λ resulted in 9 non-zero coefficients.

A total of 24 variables with *p* < 0.10 in the baseline characteristics were subjected to LASSO logistic regression model (Fig. 2a). The optimal λ resulted in 9 non-zero coefficients, which were: age (0.0146), APS III score (0.0171), hematocrit (–0.0002), ACAG (0.0245), MT (0.2250), CKD (0.0943), norepinephrine use (0.2730), vasopressin (0.6670), and coronary arteriography (–0.0935) (Fig. 2b).

**Fig. 3.**
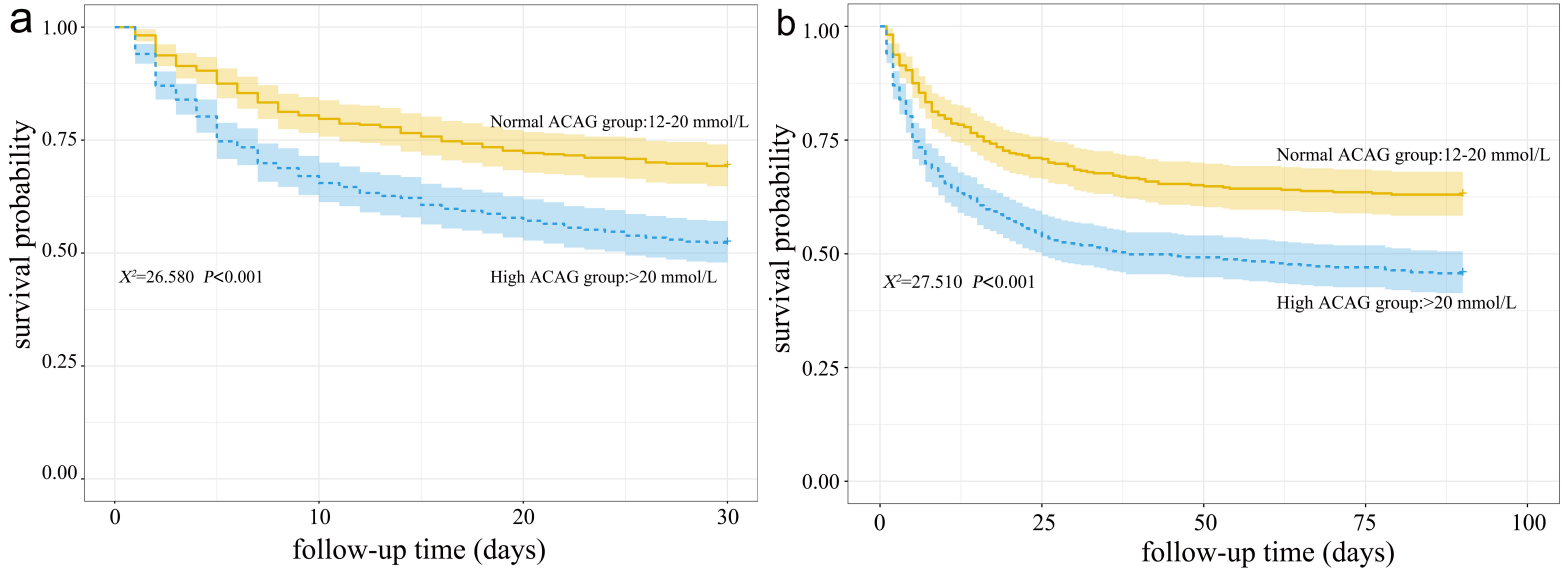
**Kaplan-Meier curves for mortality in ACAG and CS patients.** (a) 30-day outcome K-M curves. (b) 90-day outcome K-M curves. ACAG, albumin corrected anion gap; CS, cardiogenic shock.

Kaplan-Meier survival estimates curves were drawn according to the ACAG category to demonstrate the association between ACAG and 30-day and 90-day mortality in CS patients. The results showed that high ACAG was associated with 30-day mortality in CS patients, and the difference was statistically significant (log-rank test, χ^2^ = 26.580, *p* < 0.001; Fig. 3a), Meanwhile extending the survival estimates curves plotting time to 90-day we found that the difference was also statistically significant (log-rank test, χ^2^ = 27.510, *p* < 0.001; Fig. 3b).

To further demonstrate the relationship between ACAG and the risk of death in CS patients, Cox regression analysis was carried out on the relevant data. In Model 1, the relevant covariates were not adjusted. The univariate regression analysis showed that compared with the normal ACAG group, the CS patients in the high ACAG group had significantly higher mortality (30-day: HR = 1.775, 95% CI = 1.418–2.221, *p* < 0.001; 90-day: HR = 1.715, 95% CI = 1.395–2.108, *p* < 0.001) (Table 2); In Model 2, to explore whether ACAG is an independent risk factor, we controlled for other relevant covariates obtained in LASSO logistic regression model, including age, APSIII score, hematocrit, and others, and the final results revealed that ACAG was a contributing factor for mortality in patients with CS, independent of other factors (30-day: HR = 1.350, 95% CI = 1.071–1.703, *p* < 0.05; 90-day: HR = 1.321, 95% CI = 1.066–1.636, *p* < 0.05) (Table 2).

**Table 2.** **Cox proportional hazard regression analysis for 30-day and 90-day all-cause mortality**.

Group		Model 1			Model 2		
		HR	95% CI	*p* value	HR	95% CI	*p* value
30-day mortality							
	normal ACAG group	Baseline			Baseline		
	high ACAG group	1.775	1.418–2.221	<0.001	1.350	1.071–1.703	0.011
90-day mortality							
	normal ACAG group	Baseline			Baseline		
	high ACAG group	1.715	1.395–2.108	<0.001	1.321	1.066–1.636	0.011

Model 1 adjusted for nothing. Model 2 adjusted for APS III score, age, hematocrit, malignant tumor, norepinephrine use, vasopressin use, chronic kidney disease and coronary arteriography. ACAG, albumin corrected anion gap; APS, acute physiology score; HR, hazard ratio.

**Fig. 4.**
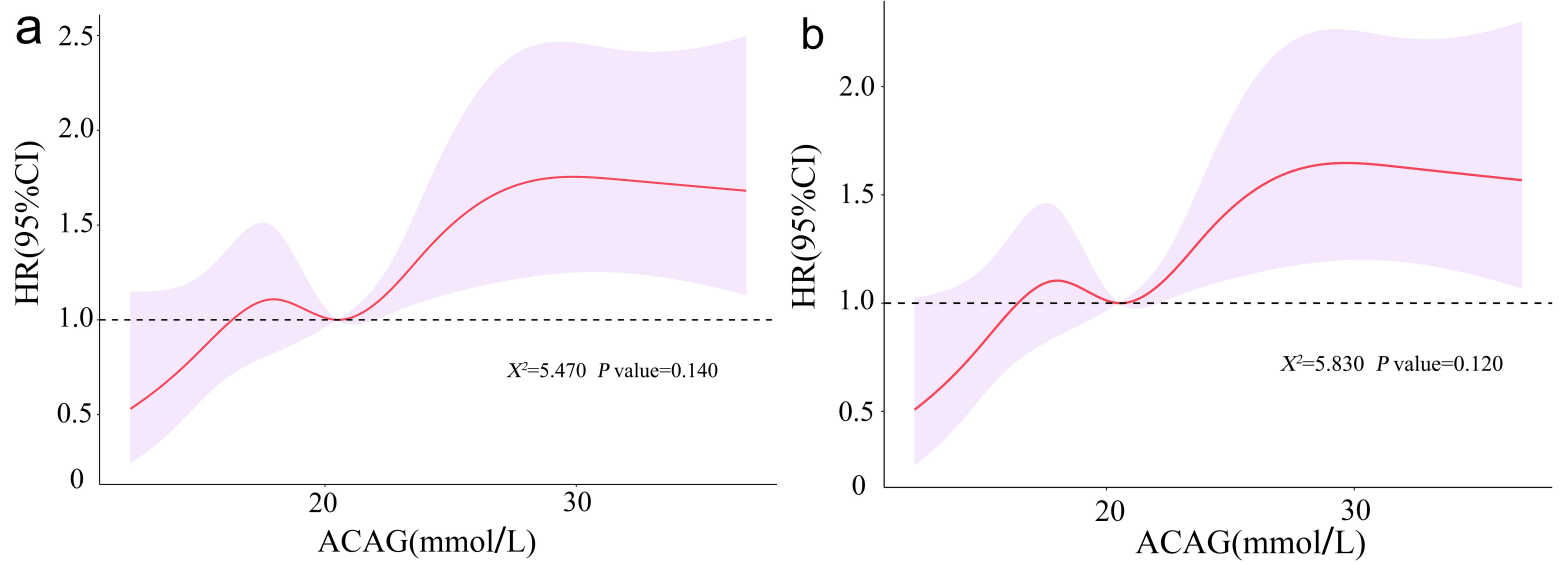
**Association between ACAG and all-cause mortality in CS patients admitted to the ICU.** (a) RCS curve for 30-day all-cause mortality. (b) RCS curve for 90-day all-cause mortality. ACAG, albumin corrected anion gap; CS, cardiogenic shock; ICU, intensive care unit; RCS, restricted cubic spline.

The RCS analysis model was established to better show the relationship between ACAG and 30 and 90 days mortality in patients with CS (30-day: Fig. 4a; 90-day: Fig. 4b). The RCS curve demonstrates a linear trend relationship between ACAG and both 30- and 90-day all-cause mortality in CS patients (30-day: χ^2^ = 5.470, *p* = 0.140; 90-day: χ^2^ = 5.830, *p* = 0.120). That is to say, with the increase of ACAG, the 30-day and 90-day all-cause mortality of these patients increases. When the ACAG increased to a certain level, the increase in the risk of death gradually flattened. When ACAG is 20.38 mmol/L, HR is about 1.

### 3.3 Analysis of ROC Curves

**Fig. 5.**
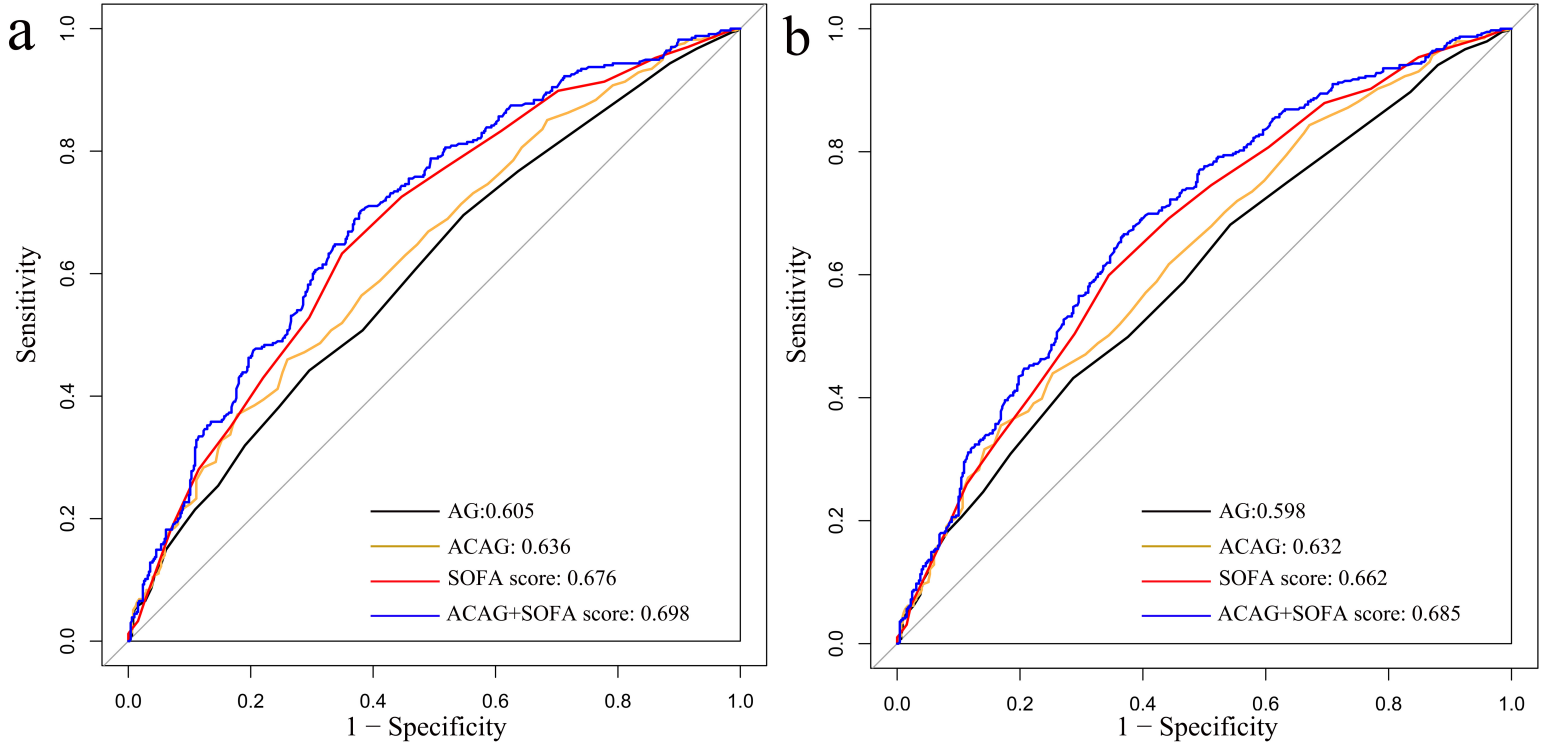
**ROC curves for predicting the mortality in CS patients.** (a) ROC curve for 30-day mortality as a study outcome. (b) ROC curve for 90-day mortality as a study outcome. AG, anion gap; ACAG, albumin corrected anion gap; SOFA, sequential organ failure assessment; CS, cardiogenic shock; ROC, receiver operating characteristics.

The ROC curve indicated that ACAG’s best cutoff value for predicting both 90-day and 30-day mortality was 22.625, with a sensitivity of 46%, a specificity of 74%, and an area under the curve (AUC) of 0.636. ACAG combined with SOFA score predicts 90-day mortality with 70.1% sensitivity and 62.1% specificity (AUC 0.698) (**Supplementary Table 1**; Fig. 5a); and it predicts 30-day mortality with 66.6% sensitivity and 63.1% specificity (AUC 0.685) (**Supplementary Table 1**; Fig. 5b).

### 3.4 Sensitivity Analysis

Since ACAG results may be affected by albumin infusion, MT, or severe liver disease, we excluded relevant patients for sensitivity analysis to verify the stability of the final results. After excluding patients who were infused with albumin 48 h before admission to the ICU, the final results showed that the link between ACAG and all-cause mortality in CS patients remained stable, either at 30 or 90 days (30-day: HR = 1.372, 95% CI = 1.084–1.735, *p* < 0.05; 90-day: HR = 1.336, 95% CI = 1.076–1.659, *p* < 0.05) (**Supplementary Table 2**). After excluding cirrhotic patients, sensitivity analysis was repeated and consistent results were obtained. The same sensitivity analyses were performed after excluding patients with MT, patients with other severe liver diseases and patients with diabetes mellitus, respectively, and the results still support our study that high ACAG (>20 mmol/L) is a stand-alone risk factor for increased 30-day and 90-day all-cause mortality in CS patients admitted to the ICU. As the potential effects of diabetes on renal function may bias the results, a sensitivity analysis of the population was performed after excluding patients with combined or concurrent diabetes. The results are also stable (All *p* < 0.05) (**Supplementary Tables 3,4**).

## 4. Discussion

Globally, CS is still the main cause of human death today, although there is a relatively mature percutaneous interventions (PCI) technology and the current research on invasive mechanical support of the heart such as veno-arterial extracorporeal membrane oxygenation (VA-ECMO) is more in-depth, and the success rate is getting higher and higher [18]. The continuously updated early classification of CS is also promoting early intervention for CS. Although everything is developing rapidly, the treatment effect is unstable due to the different severity of different individuals and the occurrence of some difficult-to-control complications. Once CS occurs, nearly half of the patients end up with death [2]. It is necessary to accurately predict the prognosis of the disease at an early stage. The hemodynamic changes in CS are extremely complex, some biomarkers such as lactate are known to have an impact on the predicted outcome of CS [19], but biomarkers for early prediction of CS mortality in clinical practice still deserve further exploration. The cardiac output is drastically reduced when a patient suffers from CS, at the same time, the perfusion of various organs in the whole body suddenly decreases, and the ensuing problem is that the balance of supply and demand of the whole body is broken, and internal environment disorder and acid-base imbalance appear. If it can not be corrected as soon as possible, the consequences will be very serious [20]. When AMI and other conditions develop into CS, most tissues already have a certain degree of acidosis due to ischemia and hypoxia. Studies have pointed out that acidosis often indicates a worse outcome of CS, and there is a relationship between it and the severity of shock [5]. Clinical evaluation of acidosis often relies on arterial blood gas analysis. It is well known that venous blood is relatively easy to obtain in clinical operations. AG is calculated from plasma sodium, potassium, chloride, and bicarbonate, it is relatively easier to obtain and has wider applicability [21]. AMI is one of the primary causes of CS, and AG higher than normal is an independent risk factor for increased mortality [10]. Upon admission to the ICU, most patients had initially elevated AG, and the mortality rate for those with elevated AG was significantly higher than those with normal AG [22] and may apply to a variety of etiologies [23]. Simultaneously, some studies have found that elevated AG levels also portend a poor prognosis for CS patients, with a higher risk of death in CS patients with high AG values compared to those with low AG levels [12], which is consistent with the data obtained in our study. However, in the ICU, because of the serious condition of the patients, many patients have hypoalbuminemia, and AG may appear pseudo-normal due to the charge of albumin. One study showed that AG decreased by 2.5 mmol/L for every 10 mg/L decrease in albumin [24]. Therefore, the use of AG to identify the etiology and type of metabolic acidosis may be inaccurate. In contrast, ACAG corrects the charge carried by albumin relative to AG, thus avoiding some bias and making a more accurate judgment of acidosis. Current research indicates associated diseases may include sepsis, acute and chronic renal failure, and diabetic ketoacidosis [13, 25], It is reasonable to believe that ACAG is the better predictor, and indeed this conjecture was confirmed in our follow-up study.

It is worth mentioning that Alb itself also has a greater role in the prediction of severe diseases. As an indicator that is easier to obtain in the early stage of clinical practice, it has been proven to be an independent predictor of the prognosis of various diseases. Some studies have found that for elderly critically ill patients, low albumin may accelerate their death process [26]. In addition, related studies on CS have shown that CS patients with hypoalbuminemia have a significantly increased risk of death [27]. It’s not the only case, our study found that non-survivors had significantly lower albumin levels than survivors. On the surface, ACAG is an organic combination of albumin and AG that can more comprehensively reflect the true level of acid-base in the body. However, in clinical practice, ACAG reflects the two pathological conditions of hypoalbuminemia and metabolic acidosis. Compared with patients in general wards, patients admitted to the ICU have greater consumption and are more prone to hypoalbuminemia. Furthermore, they are also very prone to the complication of metabolic acidosis when shock occurs [28]. It is also obvious that in our study for CS patients, non-survivors group had higher ACAG values than survivors group. In recent years, the relationship between ACAG and heart disease has been gradually exposed to the public. ACAG was shown to be a stand-alone risk factor for all-cause mortality in patients with cardiac arrest during hospitalization [29]. And one study showed that elevated ACAG levels increased the incidence of heart failure in AMI patients [30]. In the above-mentioned studies, ACAG has been shown to have a relationship with the prognosis of many heart-related diseases. Then it is worth exploring whether there is a more in-depth association between ACAG and CS patients. In our study, we found that the cumulative survival of CS patients in the high ACAG group was significantly lower than that of the normal ACAG group, and the results were consistent with the 30-day and 90-day outcome, and an elevated ACAG measured at the time of ICU admission may indeed be indicative of a poor prognosis. Meanwhile, it has been shown that ACAG has an advantage in predicting the sensitivity and specificity of recovery of autonomic circulation after CPR in patients with cardiopulmonary arrest [14]. And, elevated preoperative ACAG is also an independent risk factor for in-hospital and long-term mortality in coronary artery bypass graft patients [31]. Therefore, the aim of our study was to investigate whether ACAG is an independent risk factor for mortality in CS patients and whether it is predictive and stable.

Analysis of baseline data showed that ACAG was positively associated with the risk of death in patients with CS and negatively associated with albumin. In addition, several other laboratory-related parameters, including hematocrit, PT, BUN, and Scr, were also found to be associated with mortality risk in CS patients, and comorbidities, examination, and treatment measures may affect the final patient outcome. More patients in the non-survivors group were on blood pressure-raising drugs such as norepinephrine, and more were treated with MV and continuous renal replacement therapy (CRRT), which may be related to their more critical condition. In order to get more reliable predictors we performed the LASSO analysis model on the data of characteristics that may have an impact on the final mortality in the univariate analysis. Nine non-zero parameters, such as ACAG, were obtained by 10-fold cross-validation. To investigate further whether ACAG can be used as a predictor of mortality in CS patients, we established a Cox multifactor analysis model and found that the results were very stable after multifactor analysis with 30 and 90 days mortality as nodes, and in patients suffered from CS admitted to the ICU, ACAG was found to be an independent predictor of mortality. At the same time, the RCS curves also showed a relationship between ACAG and all-cause mortality at 30 and 90 days in CS patients, with a trend toward higher mortality as ACAG increased. Finally, it was also shown in the ROC curve that ACAG has a larger area under the curve compared to AG. And the comparative stability of ACAG in predicting mortality in CS patients admitted to the ICU was also verified in several subsequent sensitivity analyses. In conclusion, our study showed that ACAG performed well in predicting 30- and 90-day mortality in CS patients in the ICU. In the future, it may be applied clinically to guide the treatment plan of relevant patients at an early stage, and further reduce clinical mortality.

Although this study tried to avoid errors in sample collection and statistical analysis and to eliminate errors caused by changes in disease conditions, the sample values obtained when first entering the ICU were selected to calculate ACAG during sample collection, this study still has certain limitations: (1) Although the MIMIC-IV database has a large sample size, it is a single-center database, lack of diversity. Therefore, our results suffer from uniformity and may be inevitably biased. (2) Due to the singularity of the database, we can only examine the connection between ACAG and the death rate among CS patients, and the pathophysiological mechanism should be further explored in the future. (3) In addition, dynamic monitoring of changes in ACAG values in the present study was not achieved, as well as a subgroup analysis of some comorbidities that may affect the prognostic outcome may be something that should be done in further studies.

## 5. Conclusions

ACAG is an independent risk factor for 30- and 90-day mortality in CS patients and may predict poor clinical outcomes. According to our study, ACAG >20 mmol/L has a strong correlation with the prognosis of CS patients, and its predictive power is stable but its specificity is limited. Clinically, it is important to monitor CS patients with high ACAG levels to promptly correct electrolyte and acid-base imbalances. In the future, further prospective clinical studies with large samples may be needed to clarify the relationship and verify whether it has actual clinical significance.

## Data Availability

All data is extracted by those who have completed the Collaborative Institution Training Program exam and have access to the database for data extraction. (Certification No.1 LZ: 53446653 and No.2 MY: 51168595; Data site: https://physionet.org/content/mimiciv/2.0/).

## Abbreviations

ACAG, Albumin-corrected anion gap; AG, Anion gap; AKI, Acute kidney injury; Alb, Albumin; ALT, Alanine aminotransferase; AMI, Acute myocardial infarction; APS, Acute Physiology Score; AUC, Area under the curve; BUN, Blood urea nitrogen; CKD, Chronic kidney disease; CS, Cardiogenic shock; ICU, Intensive care unit; LASSO, Least absolute shrinkage and selection operator; MIMIC IV, Medical Information Mart for Intensive Care-IV; MT, Malignant tumors; PCI, Percutaneous interventions; PT, Prothrombin time; RCS, Restricted cubic spline; ROC, Receiver operating characteristics; SOFA, Sequential organ failure assessment; VA-ECMO, Veno-arterial extracorporeal membrane oxygenation; WBC, White blood cell.

## Supplementary Material

Supplementary material associated with this article can be found, in the online version, at https://doi.org/10.31083/j.rcm2506226.
