# Supplementary material for: Association between Albumin-Corrected Anion Gap and Mortality in Patients with Cardiogenic Shock
Source: Rev Cardiovasc Med. 2024 Jun 21;25(6):226. doi: 10.31083/j.rcm2506226 (PMC11270101; doi:10.31083/j.rcm2506226)
Supplement: Supplementary file 1 [file 2153-8174-25-6-226-s1.docx]

**Supplementary Table 1** Comparison of the prognostic performance of AG, ACAG, SOFA score, and the combined indicators in patients.

| Variables | AUC(95% CI) | Cut-off | Sensitivity | Specificity | PPV | NPV | PLR | NLR | *P1* | *P2* |
| --- | --- | --- | --- | --- | --- | --- | --- | --- | --- | --- |
| 30-day |  |  |  |  |  |  |  |  |  |  |
| AG | 0.605(0.566-0.643) | 16.500 | 0.696 | 0.452 | 0.458 | 0.691 | 1.270 | 0.673 |  |  |
| ACAG | 0.636(0.598-0.674) | 22.625 | 0.460 | 0.740 | 0.540 | 0.673 | 1.769 | 0.730 | <0.001 | <0.001 |
| SOFA score | 0.676(0.640-0.713) | 9.500 | 0.633 | 0.651 | 0.546 | 0.727 | 1.812 | 0.564 |  | 0.008 |
| ACAG+SOFA score | 0.698(0.662-0.734) | - | 0.701 | 0.621 | 0.599 | 0.758 | 1.851 | 0.481 |  |  |
| 90-day |  |  |  |  |  |  |  |  |  |  |
| AG | 0.598(0.560-0.637) | 19.500 | 0.432 | 0.713 | 0.566 | 0.634 | 1.252 | 0.796 |  |  |
| ACAG | 0.632(0.594-0.669) | 22.625 | 0.440 | 0.747 | 0.600 | 0.606 | 1.735 | 0.751 | <0.001 | <0.001 |
| SOFA score | 0.662(0.625-0.698) | 9.500 | 0.599 | 0.656 | 0.649 | 0.601 | 1.739 | 0.612 |  | 0.008 |
| ACAG+SOFA score | 0.685(0.649-0.721) | - | 0.666 | 0.631 | 0.609 | 0.686 | 1.805 | 0.530 |  |  |

ACAG; albumin corrects anion gap; SOFA, sequential organ failure assessment; AUC, area under the curve; PPV, positive predictive value; NPV, negative predictive value; PLR, positive likelihood ratio; NLR, negative likelihood ratio. *P1*, *P*-value for the equality compared to AG; *P2*, *P*-value for the equality compared ACAG or SOFA score alone.

**Supplementary Table 2** Sensitivity analysis after exclusion of patients with albumin infusion 48h before admission to ICU

| Group | Model 1 | | Model 2 | |
| --- | --- | --- | --- | --- |
|  | 95%CI | *P* value | 95%CI | *P* value |
| 30-days mortality |  |  |  |  |
| normal ACAG group  high ACAG group | Ref  1.441-2.270 | ＜0.001 | Ref  1.084-1.735 | 0.008 |
| 90-days mortality |  |  |  |  |
| normal ACAG group | Ref |  | Ref |  |
| high ACAG group | 1.412-2.145 | ＜0.001 | 1.076-1.659 | 0.009 |

Model 1 adjusted for nothing.

Model 2 adjusted for age, APS III score, hematocrit, malignant tumor, norepinephrine use, vasopressin use, chronic kidney disease, and coronary arteriography. ACAG, albumin corrected anion gap

**Supplementary Table 3** Sensitivity analysis of patients with malignancy, cirrhosis, and other severe liver diseases was excluded separately.

|  | Exclude Malignant tumors | | | | Exclude Cirrhosis | | | | Exclude Other serious liver diseases | | | |
| --- | --- | --- | --- | --- | --- | --- | --- | --- | --- | --- | --- | --- |
| Group | Model 1 | | Model 2 | | Model 1 | | Model 2 | | Model 1 | | Model 2 | |
|  | 95%CI | *P value* | 95%CI | *P value* | 95%CI | *P value* | 95%CI | *P value* | 95%CI | *P value* | 95%CI | *P value* |
| 30-days mortality |  |  |  |  |  |  |  |  |  |  |  |  |
| normal ACAG group | Ref |  | Ref |  | Ref |  | Ref |  | Ref |  | Ref |  |
| high ACAG group | 1.335-2.157 | ＜0.001 | 1.002-1.646 | 0.048 | 1.463-2.340 | ＜0.001 | 1.081-1.760 | 0.010 | 1.447-2.293 | ＜0.001 | 1.093-1.756 | 0.007 |
| 90-days mortality |  |  |  |  |  |  |  |  |  |  |  |  |
| normal ACAG group | Ref |  | Ref |  | Ref |  | Ref |  | Ref |  | Ref |  |
| high ACAG group | 1.325-2.061 | ＜0.001 | 1.018-1.609 | 0.035 | 1.438-2.212 | ＜0.001 | 1.081-1.691 | 0.008 | 1.4.7-2.149 | ＜0.001 | 1.079-1.671 | 0.008 |

Model 1 adjusted for nothing.

Model 2 adjusted for indicators not excluded in age, APS III score, hematocrit, malignant tumor, norepinephrine use, vasopressin use, chronic kidney disease, and coronary arteriography. ACAG, albumin corrected anion gap.

**Supplementary Table 4** Sensitivity analysis after exclusion of patients with combined or concurrent diabetes

| Group | Model 1 | | Model 2 | |
| --- | --- | --- | --- | --- |
|  | 95%CI | *P* value | 95%CI | *P* value |
| 30-days mortality |  |  |  |  |
| normal ACAG group  high ACAG group | Ref  1.549-2.759 | ＜0.001 | Ref  1.069-1.944 | 0.016 |
| 90-days mortality |  |  |  |  |
| normal ACAG group | Ref |  | Ref |  |
| high ACAG group | 1.494-2.512 | ＜0.001 | 1.063-1.824 | 0.016 |

Model 1 adjusted for nothing.

Model 2 adjusted for age, APS III score, hematocrit, malignant tumor, norepinephrine use, vasopressin use, chronic kidney disease, and coronary arteriography. ACAG, albumin corrected anion gap.
